# Chasing red herrings: Can visual distracters extend the time children take to open child resistant vials?

**DOI:** 10.1371/journal.pone.0207738

**Published:** 2018-12-12

**Authors:** Rita Chen, Nora M. Bello, Mark W. Becker, Laura Bix

**Affiliations:** 1 School of Packaging, Michigan State University, East Lansing, MI, United States of America; 2 Department of Statistics, Kansas State University, Manhattan, KS, United States of America; 3 Cognitive Group, Department of Psychology, East Lansing, MI, United States of America; University of Lincoln, UNITED KINGDOM

## Abstract

**Background:**

Unintentional exposure to medications is a noted problem in pediatric populations despite the prevalent use of child-resistant (CR) packaging and educational campaigns informing consumers about appropriate storage.

**Objective:**

Conduct a proof-of concept study that evaluates how package designs that engage the attention of children in meaningless ways affect opening time and number of openings.

**Study design:**

Non-CR vials with or without distracters were provided to 108 children (24–51 months) in pairs. Each participant was handed a vial and instructed to “do whatever you want to with it.” Successful opening and time to opening were recorded. Data were analyzed using generalized linear mixed models.

**Results:**

Older children were approximately four times more likely than younger children to successfully open a vial with a visual distracter (P = 0.049); when distracters were not present, no evidence for differences was apparent between age groups (P = 0.64). For successful openings of either age group, distracter presence significantly prolonged time to opening (P = 0.0375); vials containing distracters took nearly three times longer to open than those without.

**Conclusions:**

Existing CR designs almost exclusively rely on late stages of information processing (e.g. difficult to understand or open). Our results suggest that packaging designs that target early stage processing (i.e. perception) represent a potential paradigm for creating effective CR designs. It should be acknowledged that visual distracters, by their very nature, have the potential to act as "attractive nuisances" (i.e. if it were to be so effective that it drew children to the hazard). Further studies designed to specifically investigate this possibility are advised.

## Introduction

The Poison Prevention Packaging Act (PPPA) of 1970 defined special packaging, now referred to as “child-resistant” (CR) packaging, as,

“packaging that is designed or constructed to be significantly difficult for children under five years of age to open or obtain a toxic or harmful amount of the substance contained therein within a reasonable time and not difficult for normal adults to use properly, but does not mean packaging which all such children cannot open or obtain a toxic or harmful amount within a reasonable time.” [1]

The PPPA, and the regulations it authorizes, are credited by the staff of the US Consumer Products Safety Commission (CPSC) as saving the lives of more than 900 children since the requirements were enacted in the US in the early 1970s [2]. When the many global standards that closely mimic the US protocol are considered [3], it is likely 1,000s of children have been saved. A recent, systematic review of the state of knowledge regarding child resistant packaging concluded that the use of child-resistant (CR) packaging is associated with reductions in child mortality and recommended that requirements be expanded to other products (tobacco) to reduce poisoning among children under six years of age [4].

Despite documented reductions in the unintentional poisoning of children since the introduction of CR packaging [5, 6], unintentional ingestion of medication by children remains a serious concern. Between 2001 and 2008, more than half a million children age five or younger visited an emergency room because of unintentional exposure or overexposure to medication, with over half of these the result of accidental self-exposure to prescription drugs [7]. Indeed, pharmaceutical products have been shown to be a predominant cause of pediatric poisoning, sending one out of every 151 two-year-olds to Emergency Departments (ED) and accounting for 60% of accidental deaths in this age group [8, 9].

Three phenomena further compound and accelerate the issue. Namely:

1. *Medicine is more common around the household than ever before*. Between 1980 and 2015 prescription expenditures at US pharmacies increased from $1.4 billion to $4.0 billion [10, 11] and sales of over-the-counter (OTC) medicines increased over six-fold (from $5.5 billion in 1980 to $34.3 billion in 2017) [12].
2. *The “typical” household is changing; more and more homes are multi-generational*, *with seniors*, *characterized by higher rates of medication use*, *cohabitating with small children more than ever before*. Of the 65 million grandparents in the US in 2012, about 7 million (10%) lived with at least one grandchild, up from the 7% reported in 1992 [13]. Further, 74% of grandparents indicate taking a prescription medication every day [14]. In this same year (2012), it was reported that 2.7 million grandparents were raising their grandchildren and that about 39% of these caregivers provided care for a child under the age of five [13]. A child is seen every 8 minutes in the emergency room for poisoning due to medication [15] with 86% of emergency room visits the result of the child accessing an adult’s medication, 38% of which were grandparents [16].
3. *Children are spending greater amounts of time in closer proximity to medications*. Children are remaining indoors and engaging in more solitary and sedentary pursuits than previous cohorts of toddlers [17–19].

These factors combine to place toddlers and preschoolers in close proximity to growing numbers of medications.

## Research drivers and aims

To characterize the existing approaches taken to develop child resistant packaging and encourage new tactics for the design process, we frame our review of design approaches in de la Fuente’s Human Package Interaction Model (HPIM) [20]. The HPIM model, adapted from an information processing model presented by DeJoy [21], posits that information processing occurs in a serialized set of five steps. (See Table 1). Consider the action of opening a package with the potential for exposure to a problematic product. The child is initially exposed to the packaged product (step 1: Exposure; Table 1). Next, the child perceives the closure area using one (or more) of the five senses (step 2: Perception; Table 1) and uses cognitive resources to translate the information received from the senses into a signal that can be interpreted by the brain (step 3: encodation; Table 1). Finally, the child must comprehend/understand how the system works (step 4: comprehension; Table 1) to then engage the motor system to physically manipulate the closure for opening (step 5: action/execution; Table 1). Although a child could certainly exert random trials on the system that could result in an opening, purposeful, correct execution requires serialized processing of the information; one step followed by the next. When approached this way, the HPIM model presents a processing framework offering possible insight that can be leveraged to enhance the child resistance of packaging.

**Table 1:** Serialized steps of information processing (adapted from DeJoy (21)).

Steps related to processing	System(s) engaged	Current strategies targeting varied steps in the information processing cycle
1. Exposure to information		Educational Campaigns regarding safe storage practice (Up and Away- US;[22]); required use of a tool to open (exposure to both package and tool is necessary;[23])
2. Attention to information	Perceptual Systems	
3. Encoding of information	Perceptual and Cognitive Systems	
4. Comprehension of information	Cognitive System	Simultaneous, dissimilar motion [23]; hidden alignment [23]
5. Action based on information	Motor System	Simultaneous, dissimilar motion [23]; hidden alignment [23]; utilize an adult-sized finger or hand [3]; adult strength required [3, 23]

A recent, systematic review of medication packaging and older adults concludes that there are two primary streams of study regarding medication containers: (1) physical functionality and user capability and (2) the impact of package design on medication management [6]. Our own review of the existing tactics used to develop child resistant designs further supports the idea that packaging as a physical barrier (late stages of the processing model) is a common strategy for achieving child resistance. Specifically, we identified six broad approaches that have been leveraged in an attempt to reduce unintentional exposure of children to household medications. The approaches taken were: (1) educational campaigns (2) tools are required for opening (3) simultaneous, dissimilar motion (4) hidden alignment of components of the packaging (5) adult sized hands and/or digits (6) significant strength required for opening.

Most of the strategies we identified appear to focus on the latter stages of information processing (cognition and action;—See Table 1 Steps 4 and 5). More specifically, child resistance has been primarily realized using design features intended to confuse (e.g. tasks that require simultaneous dissimilar motions, steps in sequence or critical thinking- see Table 1, Step 4) or physically withhold children, (e.g. tasks that require dexterity, strength or size see Table 1 Step 5).

The emphasis of CR solutions that leverage late stage information processing (i.e. systems meant to segregate children from adults based on differences in cognition or physical ability- Steps 4 and 5 in processing- see Table 1) is not surprising. After all, we are attempting to physically segregate children from these products. Thus, disallowing access via physical barriers is a logical, intuitive approach. That said, the use of designs which provide complex motor actions for opening can also be problematic for older adults and frail individuals with limited strength or dexterity.

We postulate that the early portion of the model (Steps 1 and 2) provides an opportunity for developing CR strategies that prolong the time to opening in a way that is not likely to impede older adults. Further, the existing approach (i.e. packaging requiring cognitive processing or physical ability) and our approach (extending early stage processing) are not mutually exclusive; devices that capture attention could potentially be used as an added hurdle to packages that also impose a physical barrier to entry.

Given that children rely heavily on their senses to engage their surroundings, we believe that slowing children during the early steps of information processing (i.e. attention- See Table 1 step 2) to be a rich, yet untapped, area for research. “Attention begins the mind’s process of gathering information from the surrounding social and physical environment” [24]. For children, the ability to attend objects has been indicated as an important part of gaining knowledge and cognitive development [25]. “Even young infants possess expectations about physical events which helps them to better understand the properties of objects [26],” and to improve this understanding, several domains of cognitive development implore infants to engage with objects within the environment [27]. To develop understanding of how events relate to each other (i.e. cause and effect), children eight months and younger “perform simple actions to make things happen,” such as splashing in water, banging a spoon, or pushing a button to watch a figure jump out. Additionally, young children develop an understanding of spatial relationships by moving their bodies, exploring objects [17] and using trial and error to discover how things fit together.

**Fig 1:**
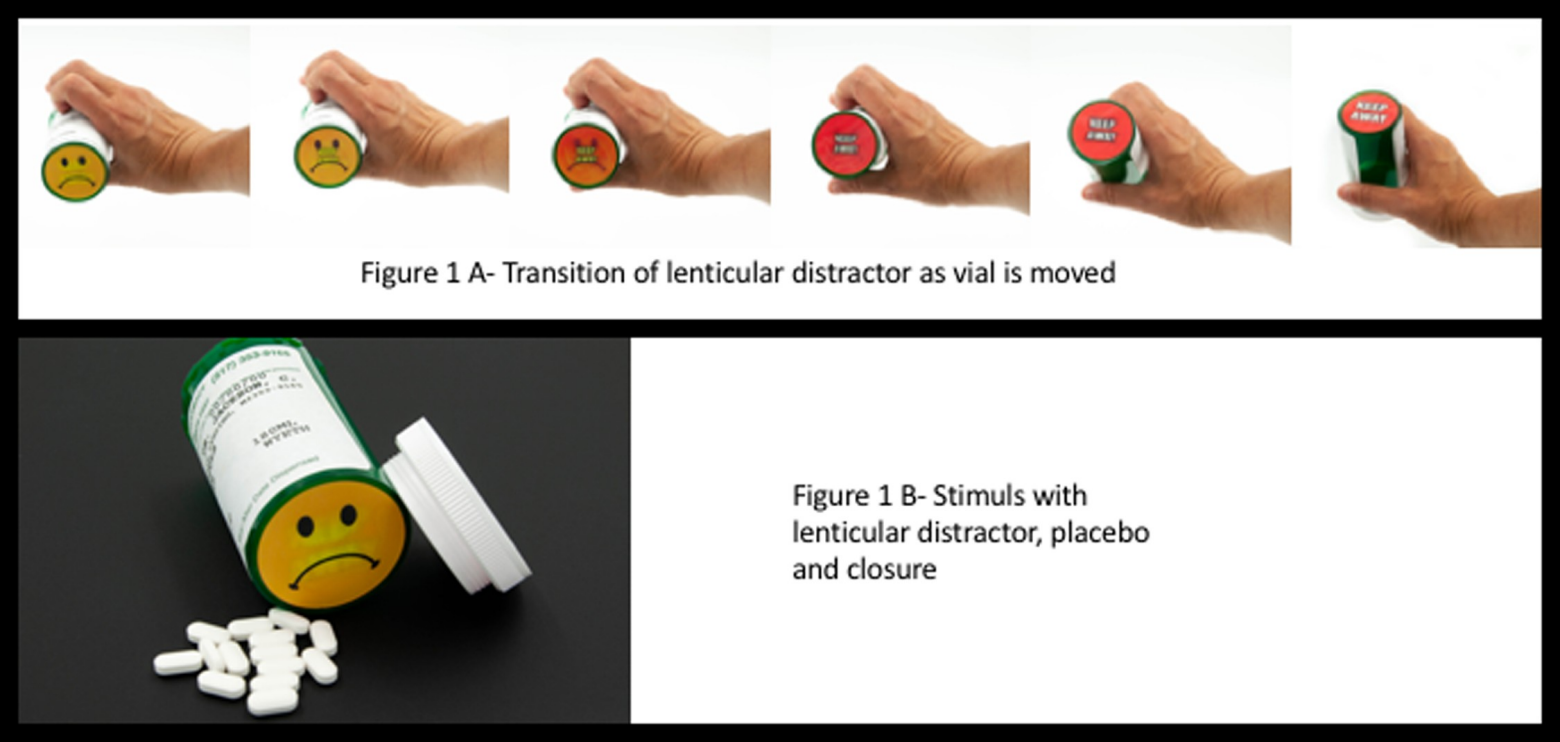
**The visual distracter consisting of a lenticular graph designed to depict a schematic facial icon (1.A, unhappy face) or warning sign (1.B) depending on perspective and motion.** Note that as the package is moved, the distracter changes in color and format from 1.A to 1.B and back.

Specifically, we postulated that by engaging children’s senses with a diversion (e.g. an intriguing visual image (See Fig 1A)) on a non-working area of the package See Fig 1B), we could extend the period of time that children were involved in early stage processing (see Table 1- Step 2); thereby extending the time to open a package.

To assess this postulate, we presented children with pharmaceutical vials outfitted with a target for their attention that we termed a “visual distracter” (see Fig 1A and 1B). Children were tested in a non-competitive context; that is, other distractions were not present [28]. A non-competitive environment represents the worst case scenario for a drug package, where nothing but the package competes for the child’s attention. The visual distracter consisted of a lenticular graphic characterized by a stereoscopic, 3D perspective that yielded the illusion of movement and depth, changing colors from yellow to red when the vial was moved (See Fig 1A).

It is a long-held belief that infants look longer at more complex stimuli than simpler stimuli, presumably because the simple stimuli are only moderately arousing, with less information to process ([28] See Cohen for a Review [29]). Our lenticular graphic was designed to be visually complicated, depicting a frowning facial icon that changes to the words “keep away” yielding the illusion of movement when viewed from different angles (Fig 1A). Design choices were based on research from the field of visual attention with adults which suggests that faces [30, 31], even schematic faces [32, 33], motion [34, 35] and color change [36] are particularly good at capturing the attention of a viewer. Although children are not studied as thoroughly, research with 3 year old children has indicated that emotional facial expressions, especially expressions of fear, can capture spatial attention [37] and looming motion and facial icons are noted to garner attention in both infants [33, 38–40] and adults [41].

## Objectives

### Overarching

Identify novel ways to prevent unintentional medication poisoning due to accidental access of package contents, with a focus on children 24–51 months of age.

### Proximal

Empirically test whether a visual distracter added to the non-working end of a package could be used to enhance its child resistant characteristics, as determined by the probability of opening success and the time to successful opening.

## Methods

All methods were approved by the Biomedical and Health Institution Review Board (BIRB) at Michigan State University under #13–246. Additionally, the study was also published in ClinicalTrials.gov under the identifier NCT01859780. Written consent was obtained from guardians and verbal assent was obtained from children.

### Participants

In order to measure the efficacy of visual distraction as a CR feature, we conducted testing with children ages 24 to 51 months. We intentionally expanded beyond the ages mandated by the US Consumer Products Safety Commission’s (CPSC) protocol for establishing a package as CR, which dictates that children aged 42–51 months of age be tested [42]. We intentionally included children younger than protocol because two-year olds represent the age group most commonly associated with calls to Poison Control Centers as reported by the National Poison Data System [14].

Standard CPSC protocol testing dictates recruitment of older children presumably because they are known to be stronger and more capable than the younger children, and, as such, represent a more robust test of the package as a physical deterrent (see Table 1- Steps 4 and 5). Additionally, when following protocol testing, the method dictates that proctors continuously encourage children to try to keep opening packages. Purposeful selection of older children, combined with the practice of continually encouraging engagement with the container during opening tests suggests the focus of current CR testing to be the late stages of processing (Table 1; Step 5). However, this approach specifically ignores early steps (Table 1; Steps 1–2) which may also influence how a CR design performs.

Participants were recruited by Great Lakes Marketing (GLM -Toledo, OH, USA), an organization that regularly conducts CR protocols with ownership of a database that lists over 600 test locations (daycares, preschools, etc.) as regular collaborators. Children were tested by researchers from MSU at the GLM facility in Toledo, OH. To be eligible to participate in the testing, subjects had to be between 24 and 51 months of age, have their guardian’s written consent and provide verbal assent to the research team. As specified in CPSC protocol testing, all children were prescreened by GLM for physical or mental impairments that could potentially impact their ability to open packages.

A total of 108 children were tested in this study. Descriptive statistics on data demographics are presented in Table 2.

**Table 2:** Demographic characterization of children recruited for this study.

	Visual distracter present on vial		Visual distracter absent from vial		Total
Age groups	24–42 months (children younger than protocol)	42–51 months^**a**^ (children of protocol age)	24–42 months (children younger than protocol)	42–51 months^**a**^ (children of protocol age)	
Subjects, #	37	17	34	20	108
Females / Males, # / #	18 / 19	9 / 8	18 / 16	9 / 11	54 / 54
Average age (min, max)	31.07m (24m, 41m)	46.29m (42.5m, 50.5m)	31.3m (24m, 41.5m)	45.72m (42m, 50.5m)	36.12m (24m, 50.5m)

^a^42-51 months is the protocol age required by the Consumer Products Safety Commission (CPSC) as required by 16 CFR 1700.20. 24–41 months is outside protocol age but has been noted to be at the greatest risk for poisoning due to unintentional ingestion of medication.

### Stimuli

For testing, we used PRX 40-dram (green) pharmaceutical vials outfitted with a reversible cap that could be secured as a push-and-turn or in a non-CR format. Caps were applied such that they were engaged in the non-CR mode and each vial was filled with 14 placebos ((See Fig 1B-lactose monohydrate excipient); placebos were white in color and intended to represent a solid oral dosage form. We secured closures in a non-CR mode because of our focus on early stage processing (Table 1 Step 2) as opposed to efficacy of the physical barrier (Table 1- Steps 4 and 5). Lenticular visual distracters (present or absent) comprised treatments.

### Experimental protocol

Federal testing for CR packaging [5], a human test intended to mimic a package being found in the home [43], mandates children be tested in pairs. This is probably because lone children tend to become shy when brought in front of a room of strange adults and asked to interact with packaging; being brought in with a partner tends to give them comfort and confidence and, as such, is a more robust test of the packaging’s ability to hold them out. This also has been noted to afford the pairs the opportunity to learn from one another, again, biasing toward a more rigorous test of proposed packaging systems, the idea being that they can observe container use within the home and learn from these observations. This is in contrast to the senior portion of the testing, which tests older adults (aged 50–70) by themselves under the assumption that they do not have someone available to demonstrate successful package use [42]. Test partners were recorded and included as clusters in the analysis.

Children and their parents were shown into one of three identical testing rooms (labeled, A, B or C) by researchers from MSU. Each child was seated on a small carpet square that was positioned in front of a screen which contained a one-way mirror through which video was recorded.

Prior to testing, a member of the research team went through a verbal assent process with the children. Children that gave an indication (verbal or otherwise, e.g. nod of head) that they did not wish to participate were excused from the study. We instructed parents, who were generally present in the testing room, to limit their involvement and comments with the following statement: “I know that parents want to help their children as much as possible, but we need to see what they do on their own. So please resist the urge to coach them or try to help them anyway."

Each child in the pair was handed a single package that was identical in treatment (i.e. distracter present or absent) to that of their test partner(s). They were then instructed, “Please do whatever you would like with this package”. It is noted that no specific instructions were given to encourage children to open their package. Testing was stopped at three minutes, or when the child had opened the package in a way that would enable access to the product, whichever came first. Successful openings and time to opening were recorded for each participant with a stop watch. Videos were reviewed post-hoc to clarify or confirm any questions regarding recorded data.

### Statistical analysis

Descriptive statistics on data demographics are presented in Table 2. A generalized linear mixed model [44] was fitted to the binary response “successful opening” (yes/no) using a Bernoulli distribution and a logit link function to connect the probability of successful opening with explanatory variables of interest. For the purpose of analysis, we characterized the subjects into two age groups: non-protocol aged children (24 < 42 months; the “young” age group) and protocol aged children (≥ 42 months of age). The linear predictor in the model considered the fixed effects of two age groups, treatment (i.e. whether the vial was outfitted with a visual distracter or not), and their 2-way interaction. The linear predictor included the random effect of test room (including testers) as an overall blocking factor and the random effect of testing pair (nested within treatment) to identify the experimental unit for treatment.

For those cases for which successful opening was accomplished, time to opening was recorded and modeled using a general linear mixed model specified to recognize the continuous nature of the response variable using a normal distribution. All other model specifications were similar to those described above. Model assumptions were checked using studentized residuals. Kenward Roger's procedure was used to estimate degrees of freedom and adjust estimates of standard errors.

All statistical models were fitted using the GLIMMIX procedure of SAS (Version 9.2, SAS Institute, Cary, NC) implemented using Newton-Raphson with ridging as the optimization technique. Estimated least square means (LSM) and corresponding standard errors (SE) or 95% confidence intervals (CI) are reported. Relevant pairwise comparisons were conducted using either Tukey-Kramer or Bonferroni adjustments, as appropriate in each case, to avoid inflation of Type I error rate due to multiple comparisons.

## Results

### Probability of opening

**Fig 2:**
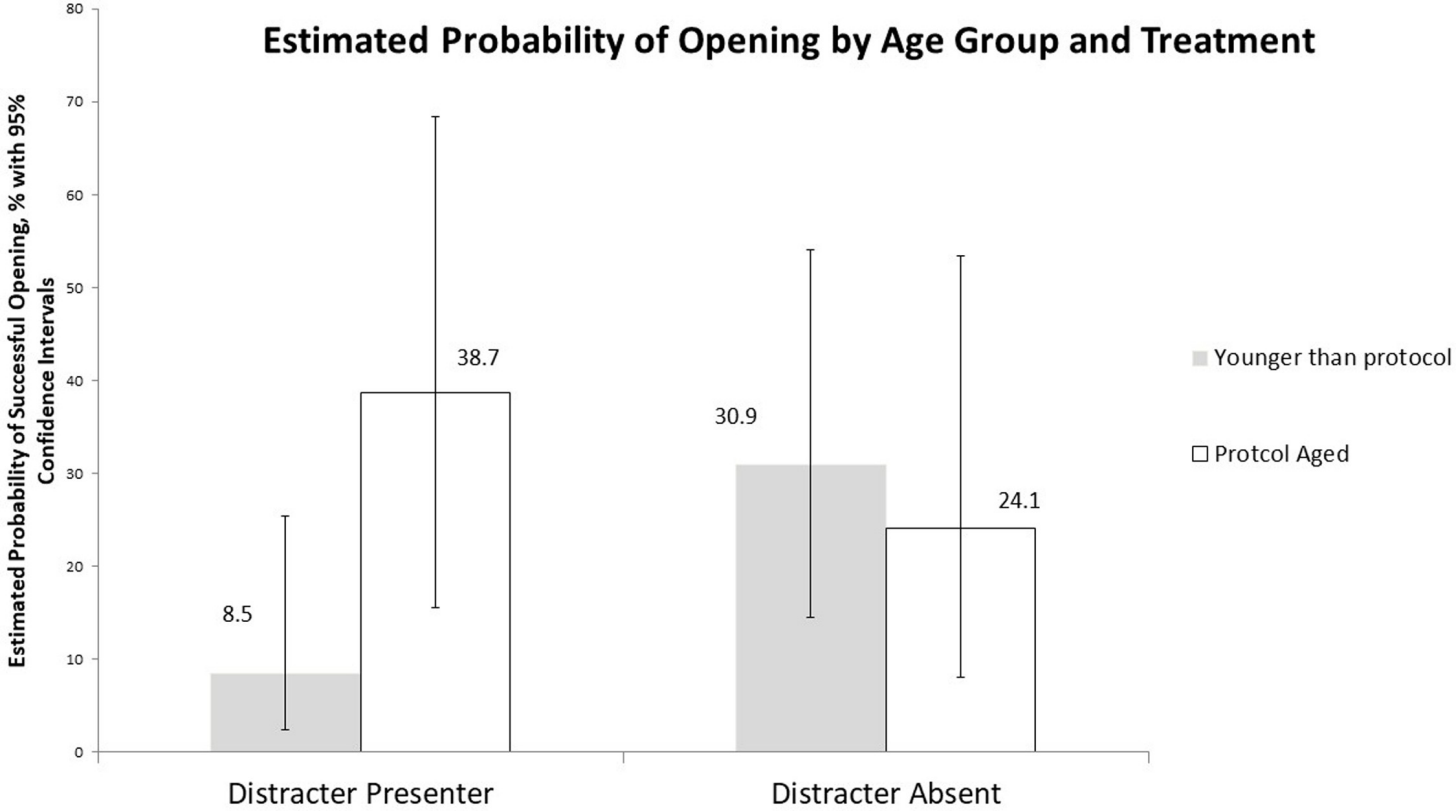
Estimated mean probability (and corresponding 95% confidence interval) of successful openings by age group for vials with or without visual distracters.

A total of 28 successful openings were recorded amongst the 108 participants tested; 25.9% of the total trials. Table 3 and Fig 2 describe the observed frequencies and estimated probability of successful openings, respectively, when visual distracters were present or absent on the vial, for both protocol-age and younger-than-protocol age children.

**Table 3:** Observed frequency of successful opening by age group for vials outfitted with or without visual distracters.

	Visual distracter present		Visual distracter absent		Total
Age groups	24–42 months (younger than protocol age)	42–51 months* (protocol age)	24–42 months (younger than protocol age)	42–51 months* (protocol age)	
# of Subjects by age group	37	17	34	20	108
Frequency of Successful openings	4	7	12	5	28 (26.0%)

Results provide evidence for a significant 2-way interaction between treatment and age group on the probability of successful vial opening (p = 0.046; Fig 2), whereby protocol-aged children (42–51 months of age) were approximately 4 times more likely than younger children to successfully open a vial outfitted with a visual distracter (LSM = 38.7%, CI = [15.6, 68.3] vs. LSM = 8.5%, CI = [2.5,25.4], respectively; p = 0.049). In turn, when visual distracters were absent from vials, no evidence for age group differences in opening was apparent (p = 0.64). Within either of the age groups, there was no evidence for an effect of visual distracter on the probability of opening the vial (p>0.10).

### Time to opening

**Fig 3:**
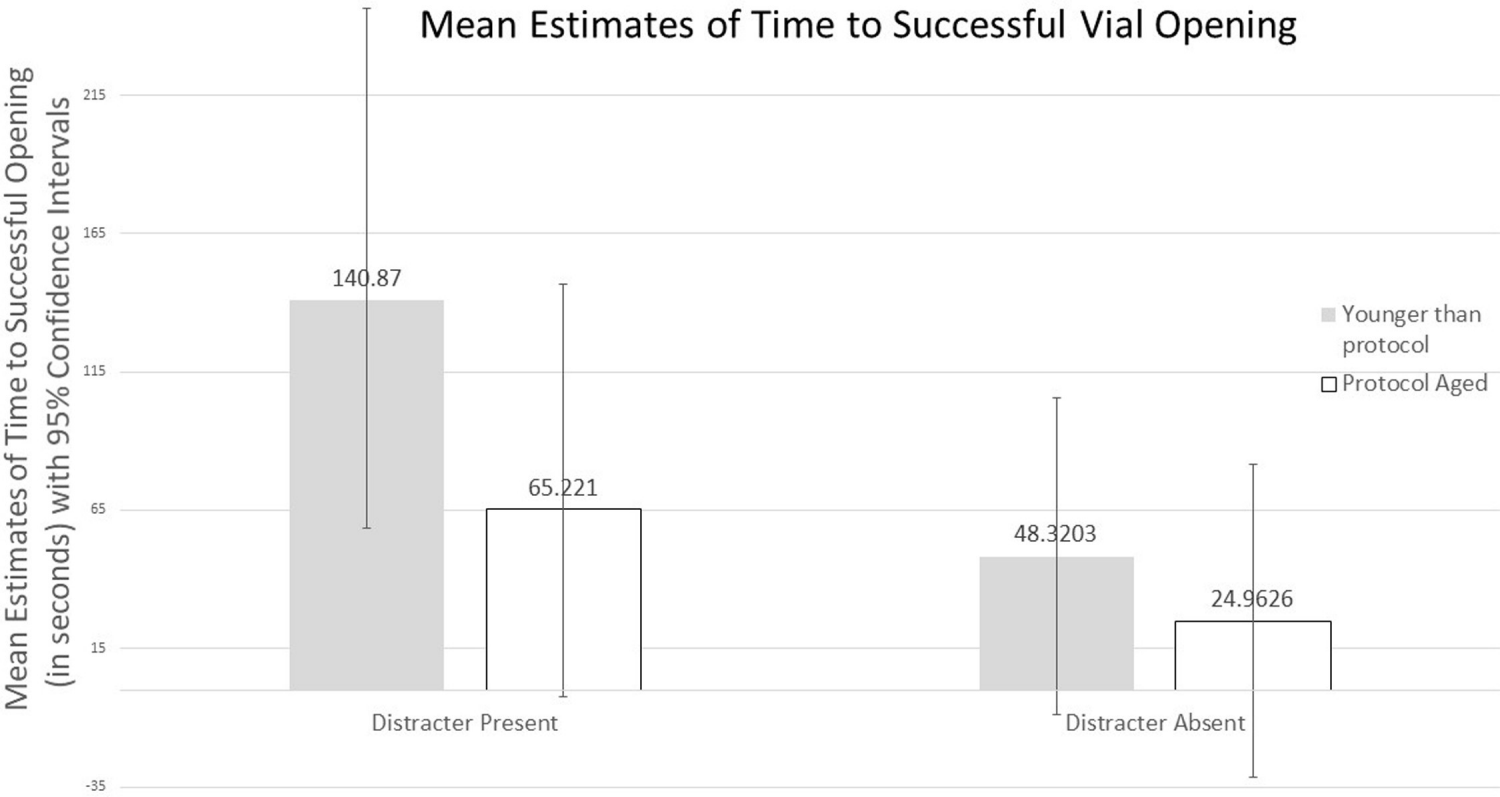
Estimated mean time to opening for successful openings by age group (and corresponding 95% confidence interval) for vials with or without visual distracters.

We also analyzed the time that it took participants to successfully open a vial for the 28 trials that comprised successful openings (See Fig 3). Results indicate evidence for a main effect of both treatment (p = 0.038) and age group (p = 0.036) on time to successfully open a vial, and no evidence for any 2-way interaction was detected (p = 0.24). Regardless of whether the vials were outfitted with visual distracters or not, older children that opened the vials did it significantly faster (LSM = 45.1 seconds; 95% CI = [36.6, 152.6]) than their younger counterparts (LSM = 94.6 seconds; 95% CI = [21.4, 111.6]). Additionally, in both age groups, children took significantly more time to open vials outfitted with visual distracters (LSM = 103.1 seconds, 95% CI = [37.0, 169.1]) than those without (LSM = 36.6 seconds, 95% CI = [21.1, 94.4]).

## Discussion

The Federal government (US) has been careful to avoid the term “child-proof,” favoring the term “child resistant” instead. Under the US construct, CR packaging is not designed to completely eliminate access to medications and other household chemicals, but is more of a “last line of defense” in a series of hurdles intended to impede access [45]. Hence, the verbiage in the law defining CR packaging, “…does not mean packaging which all such children cannot open or obtain a toxic or harmful amount within a reasonable time.” [1] This appears to be a shared global perspective; the World Health Organization’s (WHO’s) *World Report on Child Injury Prevention* indicates, “even safe packaging cannot compensate for unsafe storage.”[46]

This thinking, combined with: research which suggests the typical approach to child resistance to be a physical barrier [6, 47]; the ubiquitous presence of the push and turn closure in the US since its introduction in the 1970s; and the fact that children are developmentally predisposed to explore objects using their senses to gain an understanding of the world [17, 25–27], led us to try to think more creatively about the development of child resistant packaging. In doing so, we test the idea that a distractive device could extend the time to opening and, therefore, delay unintended exposure to potentially toxic substances without adding significant physical hindrance for older adults in need of access to container contents.

Our results are an encouraging first step. Our inference on probability of successful opening suggests that children from the younger age group (24–41 months of age), those who tend to be at the highest risk for problems associated with unintentional exposure to medications [8, 9], may be particularly receptive to visual distracters as CR features, relative to older kids (see Fig 2). Specifically, older children were significantly more likely than the younger group to open packages that contained distracters (p = 0.049). However, when distracters were absent, no age-related differences were detected (p = 0.64). That said, the evidence did not support any increase in probability of successful opening driven by presence of a visual distractor in either age group (P>0.10). Given the sample sizes, which were limited by difficulties recruiting this vulnerable audience and constrained resources to do so, further research is needed. More encouragingly, for children that successfully opened vials, openings for both age groups took significantly longer when a distracter was present (103.1 seconds 95% CI = [37.0,169.1] vs 36.6 seconds, 95% CI = [21.1,94.4]). That said, readers are cautioned. It should be acknowledged that visual distracters, by their very nature, have the potential to act as "attractive nuisances" (i.e. if it were to be so effective that it drew children to the hazard). Further studies designed to specifically investigate this possibility are an imperative prior to practical application.

## Conclusion

Results presented here encourage us to expand our archetype beyond the idea that child resistant packaging serves only as a physical barrier to entry. They implore translational research which applies fundamental knowledge from varied and disparate fields (e.g. visual perception, child development, ergonomics, and neurology) in meaningful applications to solve real-world problems creatively.

## Limitations

Our sample sizes were largely dictated by practical realities, as opposed to experimental ideals. We recruited and tested 108 children between the ages of 24 and 51 months of age for this study. To inform our sample size, we referred to the Federal test protocol (16 CFR 1700), which mandates children are tested in a series of sequential panels of 50; we had the resources available to recruit and test two full panels. Recruiting a Federally protected group (children) for this type of work (exposing children to packaging that normally contains product that they should be protected from), is quite challenging. Not only do the IRBs (rightly) require informed consent from the parents/guardians of this protected class, whom must consent to allowing their children to be exposed to child resistant containers, we must also obtain the verbal assent of the children. Our experience suggests that contracting with a commercial testing facility that has established relationships with daycares and a long history of this type of testing is the only way to be efficient. The established relationships (and record of no incidents) of these entities ease the mind of all involved and results in a much more productive recruitment effort. These contractors come at a cost, and the fact that this project was internally funded limited the number of panels we were able to recruit. As such, it is not completely clear whether null results are indicative of no effect or the study being under powered. Because the vast majority of testing of this type is done for commercial purposes; we were unable to identify a publicly available data set to perform power calculations in advance of the study. By making this data set available, it can now be used to inform future studies, which we believe to be imperative.

A considerable limitation of the study involved parental involvement. Although our protocol specified that testers instruct parents not to guide or help their children during testing, parents occasionally provide encouragement through verbal and nonverbal forms.

In the interest of full disclosure, authors would like to report that during this study, additional children pairs (other than those presented herein) were provided with a blister package, whereby visual distracters were present or absent, in a similar experimental design as described here for vials. However, we encountered unanticipated technical challenges with the design of the blister that confounded our effects of interest and prevented sound interpretation of the data. As such, data collected on blister packages was removed from further consideration and are not reported here.

## Supporting information

S1 Table Raw data in flat file. (XLSX)

## Acronyms

BIRB: Biomedical Institutional Review Board at Michigan State University
CDC: US Centers for Disease Control and Prevention
CFR: Code of Federal Regulations
CPSC: US Consumer Product Safety Commission
CR: Child Resistant
ED: Emergency Department
MSU: Michigan State University
PPPA: Poison Prevention Packaging Act
